# Cyclosporin A-related cerebral venous sinus thrombosis: A case report

**DOI:** 10.1097/MD.0000000000011642

**Published:** 2018-08-03

**Authors:** Fengjuan Gao, Jun Zhang, Fang Wang, Xiaoyan Xin, Dujuan Sha

**Affiliations:** a Department of Emergency, Nanjing Drum Tower Hospital Affiliated to Nanjing University Medical School; b The State Key Laboratory of Pharmaceutical Biotechnology, Nanjing University; c Department of Radiology, Nanjing Drum Tower Hospital Affiliated to Nanjing University Medical School, Nanjing, Jiangsu, China.

**Keywords:** cerebral venous sinus thrombosis, cyclosporin A, headache

## Abstract

**Rationale:**

Cerebral venous sinus thrombosis (CVST) is a complex life-threatening condition, and its etiology is not well understood. Although oral cyclosporin A is not a common cause of the symptoms related to CVST, there is limited information available.

**Patient concerns:**

In this study, we report a rare case of CVST in a 44-year-old woman with aplastic anemia, who was given cyclosporin A orally for a period of 18 months. She had experienced a headache for 20 days.

**Diagnoses:**

The patient was diagnosed with CVST by computed tomography venography.

**Interventions:**

Low molecular heparin (enoxaparin, 4000 AXaIU, subcutaneous injection, once every 12 hours) was administered for anticoagulation.

**Outcomes:**

The patient developed no recurrence of thrombosis during the 13-month follow-up period.

**Lessons:**

Clinicians should be aware of the possibility of CVST when patients are treated with cyclosporin A and have symptoms such as headaches.

## 1 Introduction

The etiology of cerebral venous sinus thrombosis (CVST) is complex, involving hereditary, and acquired factors, according to a report by Alvis-Miranda et al.^[1]^ CVST due to hereditary factors is rare, while CVST resulting from acquired factors is commonly observed. Acquired factors mainly include brain tumors, brain trauma, infection of the nervous system, immune system diseases, and drugs. Oral contraceptives are the most common drugs responsible for causing CVST.^[2]^ Although rare, other drugs, such as cyclosporin A and tacrolimus, have been reported to cause CVST.^[3]^ Here, we report a rare case of CVST in a 44-year-old woman, who was administered cyclosporin A orally for 18 continuous months.

## 2 Case report

**Figure 1:**
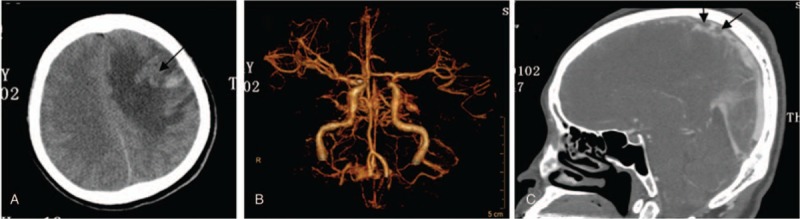
The high-signal image of the computed tomography scan shows ischemia and hemorrhage in the left frontal lobe (black arrow, A); no abnormity in cerebral arteries (B); and sinus thrombosis observed in the superior sagittal sinus by computed tomography venography (black arrow, C).

A 44-year-old woman presented to our hospital with symptoms of a headache for 20 days and weakness in the right limbs for 1 day. She had a history of aplastic anemia (AA) and had been taking 150 mg oral cyclosporin A (twice a day) continuously for 18 months. Physical examination was performed and the following parameters were noted: height, 170 cm; weight, 68 kg; body temperature, 36.5°C; and blood pressure, 142/83 mm Hg. No abnormality was observed on cardiopulmonary or abdominal examination. Neurological examination revealed conscious mind and motor aphasia; bilateral eye movement was flexible; size of the bilateral pupils were equal at 3 mm in diameter; light reflex was observed; the right nasolabial sulcus was shallow; tongue was in the middle; gag reflex was noted; neck was soft; according to the manual muscle test, the strength level of the right upper and lower limb muscles was 4; the strength level of the left upper and lower limb muscles was 5 (normal); muscle tension in all limbs was normal and physiological reflex was noted; and the Babinski sign on the right side was positive. The patient had no history of diabetes, hypertension, hyperlipidemia, liver cirrhosis, smoking, use of contraceptive pills, pregnancy, puerperium, and infection. Laboratory examination revealed: white blood cells, 6.19 × 10^9^/L (normal reference value 4–10 × 10^9^/L); neutrophils, 86.9% (normal reference value 50–70%); hemoglobin, 89 g/L (normal reference value for adult female 110–150 g/L); platelets 55 × 10^9^/L (normal reference value 100–300 × 10^9^/L); blood concentration of cyclosporin A (valley concentration), 240.7 μg/L (normal reference value 150–250 μg/L); plasma D dimer, 31.38 mg/L (normal reference value < 0.5 mg/L); and normal levels of blood protein S, protein C, anticardiolipin antibody, immune indexes, indexes of tumor, antithrombin III blood homocysteine, and blood fibrin. Ischemia and hemorrhage were observed in left frontal lobe by computed tomography (CT) scan (Fig. 1A). There was no abnormity in the cerebral arteries (Fig. 1B) and sinus thrombosis was observed in the superior sagittal sinus region with computed tomography venography (CTV) (Fig. 1C). The patient was administered low molecular heparin (enoxaparin, 4000 AXaIU, subcutaneous injection, once every 12 hours) for anticoagulation. After 2 weeks, it was replaced by warfarin, and the dosage of warfarin was adjusted by the international normalized ratio (2.0–2.5). Cyclosporin A was stopped immediately and replaced by Testosterone Undecanoate. After 30 days, the patient had no more thrombosis. But her hemoglobin concentration was declined to 55 g/L. According to the hematology specialists and neurologists’ comments, cyclosporine A was resumed again and warfarin was taken together. The dosage of cyclosporine A was adjusted between 100 to 150 mg (twice a day) according to the blood concentration. The patient developed no recurrence of thrombosis during the 13-month follow-up.

## 3 Discussion

CVST is a type of cerebrovascular disease, the incidence of which is <1% among all stroke cases.^[4]^ It has been reported that the main risk factors for CVST include oral contraceptives, pregnancy, postpartum complications, trauma, and a hypercoagulable state.^[5]^ In addition to oral contraceptives, CVST can be caused by other drugs, such as immunosuppressants and cortical hormones^[3,6–27]^ (Table 1).

**Table 1:**
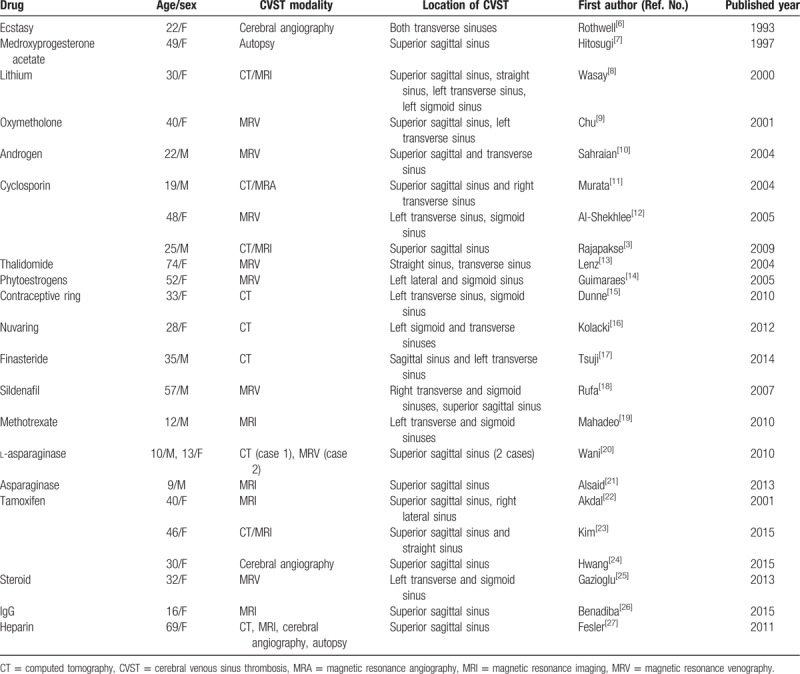
Drug factors associated with cerebral venous sinus thrombosis.

Headache is the most common clinical manifestation of CVST. Furthermore, seizures, subarachnoid hemorrhage, intracranial hypertension, and coma have been noted as symptoms in a few patients.^[28–30]^ Since the clinical manifestations of CVST lack specificity, misdiagnosis or underdiagnosis commonly occurs in clinical practice. Currently, cerebral magnetic resonance venography (MRV) is the best diagnostic method for CVST.^[31,32]^ In addition, the use of cerebral CTV is feasible, if the patient is unfit to undergo MRV. In the initial stage in our case, headache and vomiting occurred; however, no abnormal symptoms were noted on physical examination. In addition, cerebral CT or cerebral magnetic resonance imaging findings were normal. Cerebral CTV aided in the final diagnosis. Thus, for patients who are suspected to have CVST, it is suggested that cerebral MRV or CTV is performed at the earliest opportunity.

CVST can also occur as a secondary disease after intracerebral hemorrhage; this is one of the reasons for the misdiagnosis of CVST. The cerebral CT scan of our patient revealed intracerebral hemorrhage; however, on CTV, it was soon discovered to be a result of CVST. Therefore, anticoagulant therapy was promptly administered. According to the 2010 European Federation of Neurological Societies guidelines,^[4]^ intracerebral hemorrhage is not a contraindication of anticoagulant therapy in CVST. Although CVST itself can be serious, the use of anticoagulant drugs can also aggravate intracerebral hemorrhage. Furthermore, to safely perform anticoagulant therapy in patients with early intracerebral hemorrhage associated with CVST, the therapeutic decision should be individualized to the patient and caution should be taken.^[33]^

Cyclosporin A, a specific immune inhibitor, is now recognized as the first-line drug in the treatment of AA in adults. There are few reports on CVST induced by cyclosporin A,^[3,11,12]^ its mechanism is remains unclear. According to the medical history and examination results, the patient had no cerebral trauma, intracranial surgery, central nervous system infection, facial abscess, tumor, autoimmune disease, other oral drugs, puerperium, pregnancy, hypercoagulable state, or hereditary diseases; therefore, other possible causes of CVST had been ruled out. Anemia is the main symptom of AA. Anemia can also predispose to CVST. But it was reported^[34,35]^ only a weak association with severe anemia (hemoglobin < 5.0 mmol/L for nonpregnant women) and no association with mild anemia. In this patient, the hemoglobin concentration is 89 g/L (5.52 mmol/L). Thus, we consider the patient to have had CVST induced by Cyclosporin A. Cyclosporin A inhibits the release of prostacyclin from vascular endothelial cells, activates the renin–angiotensin system, increases the synthesis of systolic vascular endothelia and thromboxane A2, and increases production of free radicals; thus, this directly leads to endothelial injury and vasomotor function damage.^[36]^ In addition, cyclosporin A can activate the endogenous coagulation pathway^[37]^ and induce the expression of inducible nitric oxide synthase.^[38]^ These factors are considered to be the main mechanisms through which thrombosis is induced by cyclosporin A. According to the literature report, the time ranges of cyclosporine A induced CVST is from 9 days to 11 months,^[3,11,12]^ and our patient had taken cyclosporine A for 18 months. Therefore, the specific time for cyclosporine A induced CVST is unclear. We also noticed that the time of intravenous cyclosporin A induced CVST is shorter than that of oral cyclosporine A.^[3,11,12]^ The fastest time of the former is 9 days.^[12]^ Whether it is related to the rapid increase of blood concentration of intravenous cyclosporin A, it is not reported up to present. Patients should be informed about the possibility of CVST and caution should be taken for patients who experience headaches when being treated with oral cyclosporin A. Early diagnosis is important, such that prompt therapy can be administered to improve the prognosis of patients and reduce mortality and morbidity.

## Acknowledgments

The authors thank the assistance from our colleagues at the Department of Hematology of Nanjing Drum Tower Hospital. The authors thank Editage (www.editage.com) for English language editing.

## Author contributions

**Data curation:** Jun Zhang, Fang Wang.

**Methodology:** Xiaoyan Xin.

**Writing – original draft:** Fengjuan Gao.

**Writing – review and editing:** Dujuan Sha.
